# Practitioner Perspectives on Strategies to Promote Longer-Term Benefits of Acupuncture or Counselling for Depression: A Qualitative Study

**DOI:** 10.1371/journal.pone.0104077

**Published:** 2014-09-08

**Authors:** Hugh MacPherson, Liz Newbronner, Ruth Chamberlain, Stewart J. Richmond, Harriet Lansdown, Sara Perren, Ann Hopton, Karen Spilsbury

**Affiliations:** 1 Department of Health Sciences, University of York, York, United Kingdom; 2 Firefly Research & Evaluation, North Yorkshire, United Kingdom; Women's Hospital, School of Medicine, Zhejiang University, China

## Abstract

**Background:**

Non-pharmacological interventions for depression may help patients manage their condition. Evidence from a recent large-scale trial (ACUDep) suggests that acupuncture and counselling can provide longer-term benefits for many patients with depression. This paper describes the strategies practitioners reported using to promote longer-term benefits for their patients.

**Methods:**

A qualitative sub-study of practitioners (acupuncturists and counsellors) embedded in a randomised controlled trial. Using topic guides, data was collected from telephone interviews and a focus group, altogether involving 19 counsellors and 17 acupuncturists. Data were audio recorded, transcribed verbatim and analysed using thematic content analysis.

**Results:**

For longer-term impact, both acupuncturists and counsellors encouraged insight into root causes of depression on an individual basis and saw small incremental changes as precursors to sustained benefit. Acupuncturists stressed the importance of addressing concurrent physical symptoms, for example helping patients relax or sleep better in order to be more receptive to change, and highlighted the importance of Chinese medicine theory-based lifestyle change for lasting benefit. Counsellors more often highlighted the importance of the therapeutic relationship, emphasising the need for careful “pacing” such that the process and tools employed were tailored and timed for each individual, depending on the “readiness” to change. Our data is limited to acupuncture practitioners using the principles of traditional Chinese medicine, and counsellors using a humanistic, non-directive and person-centred approach.

**Conclusions:**

Long-term change appears to be an important focus within the practices of both acupuncturists and counsellors. To achieve this, practitioners stressed the need for an individualised approach with a focus on root causes.

## Background

Depression is a common illness in primary care. Some of the optimism regarding the potential of selective serotonin re-uptake inhibitors (SSRIs) as a treatment for depression has faded in the light of concerns about effectiveness and safety. [1] A recent meta-analysis has concluded that antidepressants have only a modest advantage over placebo, with the magnitude of benefit increasing with severity of depression. [2] There is an ongoing interest, especially among patients, in the potential of non-pharmacological treatments for depression, with the hope that they might avoid some of the concerns about antidepressants regarding safety and dependency. [3] Furthermore, for many patients, depression is a chronic and recurring illness, and non-pharmacological treatments that seek to improve longer-term outcomes are potentially of interest.

Evidence from a recent large-scale trial (ACUDep) [4] has suggested that acupuncture and counselling can provide longer-term benefits for many patients with on-going depression in primary care. The ACUDep trial recruited 755 patients with depression via 27 participating primary care practices; 302 were randomised to up to 12 acupuncture sessions; 302 to up to 12 counselling sessions; and 151 to usual care alone. The counselling was delivered within a recognised, manualised competency framework developed by Roth et al. at the University College of London's Centre for Outcomes Research and Effectiveness. [5] The acupuncture diagnosis and treatment, including selection of points, was based on the principles of traditional Chinese medicine, the details of which are reported elsewhere. [6]

Both acupuncture and counselling were found to be effective in reducing the symptoms of depression when compared to usual care, and these differences were significant at the 3 and 6 month time points, as well as when averaged over the 12 month period. [4] [7] The trial focused on the effectiveness and cost effectiveness of the interventions. However, we were keen to understand the experiences of those providing treatments (acupuncture or counselling) within the trial, and in particular the aspects of these interventions that practitioners perceived to be associated with their longer-term benefit. In doing so, these insights will help understanding of the interventions and help inform both clinical practice and any future research design.

Underreported within randomised controlled trials are qualitative analyses of strategies employed by acupuncturists and counsellors within treatment for depression that are intended to promote longer-term change. In one qualitative study, interviews with patients receiving counselling in routine practice were used to identify the key characteristics of the intervention that were experienced as beneficial in the longer term. [8] In the authors' interpretation, a key component was the client's active engagement during and between counselling sessions. In turn this enabled a change to take place in the way they conducted their lives and relationships. We are unaware of any published qualitative studies of the processes of care associated with long-term change in the case of acupuncture for depression.

This qualitative study, embedded within a clinical trial, was designed to explore the perceptions and experiences of counsellors and acupuncturists who provided the treatment interventions within the trial. The aim was to understand better the key aspects of their work and their perceptions of the ‘active ingredients' of counselling or acupuncture that were intended to promote longer-term benefits for the patients and may help in our understanding of the trial results. The study also provided an opportunity to identify important issues relating to professional practice and future policy, which are not reported here, including support needs and working arrangements; the acceptability and feasibility of different treatment options; the implications for the provision of acupuncture or humanistic counselling for treating depression within primary care settings; and related commissioning issues.

## Methods

### Recruitment of participants

To provide treatments for trial participants, 41 counsellors and 23 acupuncturists were recruited from across the regions of Yorkshire and the North East of England. Altogether these practitioners treated 497 (out of 604) patients participating in the intervention arms of the ACUDep trial. After the end of the trial's treatment phase, acupuncturists and counsellors who had consulted with at least two patients in the trial were invited to take part in the qualitative sub-study. They were sent an information sheet and consent form and asked to indicate whether they would prefer to be involved in a one-to-one telephone interview or a focus group. The aim was to recruit up to 30 counsellors and acupuncturists (approximately 15 in each group). Ethical approval was obtained from the York NHS Research Ethics Committee (REC ref: 09/H1311/75), which included approval for procedures that required written consent from all participants.

### Interview and focus group data collection

The topic guides for the interviews and focus group were developed by the ACUDep research team to address the main research question of practitioner strategies intended to promote longer-term benefits for patients – a key finding of the trial results. The initial ideas were developed into topic guides for the interviews, one for each of the practitioner groups, and one for the focus group discussion which comprised both acupuncturists and counsellors. Along with questions, the topic guides also included prompts.These were then refined further at face-to-face meetings, such that the guides covered the practitioner's professional background and experience of working with depression, their approach to treating depression, their perceptions of working within the trial, and their thoughts on the implications for policy and professional practice. These issues were considered important for interpretation of the qualitative data.

All participants provided written consent. Those who agreed to take part in a telephone interview were contacted (either by telephone or email) to arrange a convenient time for the one-to-one interview. Arrangements were confirmed by email and the topic guide to be used in the interview was attached. Only small numbers of practitioners expressed a preference for a focus group discussion and so only one group discussion was held. Many issues covered in the group discussion were similar to those expressed in the individual interviews. However, the group discussion provided an opportunity for therapists to explore together any differences and similarities in their perceptions and experiences. This added to the richness of the data. The interviews and focus group were conducted by two experienced health services researchers from the research team who were not involved in the conduct of the trial itself (LN and RC). The interviews commonly lasted around 45 minutes (range 40 to 90) and the focus group 90 minutes. They were all audio-recorded (with participants' permission) and transcribed verbatim.

### Data analysis

The transcripts were analysed thematically. [9] The main areas of inquiry covered in the topic guides were used for a framework approach to thematic content analysis, which involved analysing the data across the two groups of practitioners contributing to the study. [10] The framework was created in an Excel spreadsheet and coded and populated with data from the transcripts by the two researchers who had conducted the interviews (LN and RC). An inductive process was used to identify themes, drawing on shared experiences or points of difference identified from the data. As this study was intended to broaden our understanding of practice in relation to the treatment of patients with depression, the analysis also sought to draw out findings which might inform the practice of acupuncturists and counsellors.

## Results

### Practitioner characteristics

Forty-one therapists consented to be involved in the study but five therapists later withdrew or didn't respond when the fieldwork was being set up. The sub-study included individual telephone interviews with15 counsellors and13 acupuncturists (n = 28) and one focus group with 4 counsellors and 4 acupuncturists (n = 8) (Table 1). The sample for the qualitative sub-study included over half (56%) of the total number of therapists involved in the main trial.

**Table 1:** Breakdown of study participants.

	Number involved in main trial	Number involved in one-to-one interviews	Number involved in the focus group	Total number participating
Counsellors	41	15	4	19
Acupuncturists	23	13	4	17
Total	64	28	8	36

The 17 participating acupuncturists were registered members of the British Acupuncture Council with at least three years' post-qualification experience and practising with the theoretical approach of traditional Chinese medicine. [6] The acupuncturists were predominantly male (39% were female), with an average duration of practice of 12 years. In the trial they treated on average 9 patients, who attended on average for 10 sessions. The 19 counsellors were members of the British Association for Counselling and Psychotherapy, who were accredited or were eligible for accreditation having completed 400 supervised hours post-qualification, and provided clients a humanistic style of counselling based on competences developed for Skills for Health. [11] They were predominantly female practitioners (79%), with an average duration of practice of 7 years. In the trial they treated on average 7 patients, who attended on average for 9 sessions.

### Trial patient characteristics

Practitioners were treating patients in the trial who had ongoing depression for at least 3 months and a score of 20 or more on the Beck Depression Inventory (BDI-II), with 38% having depression categorised as “moderate” and 62% “severe”. [4] The mean age was 44 years and 73% were female. They were (on average) 25 years old when they experienced their first episode of depression. In terms of prescribed medication, 69% were on anti-depressants and 48% were on analgesics.

### Overview of the findings from thematic analysis

A cluster of eight themes emerged from the framework analysis. Almost all of the acupuncturists and counsellors stressed the importance they attached to promoting longer-term benefits. To achieve this, practitioners commonly reported on the need to focus on the root causes, to address treatment for each individual patient, and to value small incremental changes from one session to the next as precursors to sustained benefit over the longer term. Acupuncturists more commonly reported on the need to address concurrent physical symptoms, for example helping patients relax or sleep better in order to be more receptive to change. Most also highlighted the importance of Chinese medicine theory-based lifestyle change as an approach to promoting more lasting benefit. Counsellors more often highlighted the importance of the therapeutic relationship, emphasising the need for careful “pacing” such that the process and tools employed were tailored and timed for each individual, depending on the “readiness” to change. These findings are presented in more detail below.

### Importance of a long-term focus

Long-term change appears to be an important focus within the practices of both acupuncturists and counsellors. Almost all of the practitioners mentioned the long-term perspective as inherent to the way they worked.

*with something like depression, longer term for me, longer term approach is very fundamental*.(acupuncturist1)

For acupuncturists, this perspective was drawn from the theoretical approach of traditional Chinese medicine, in which long-term change needed to be facilitated by lifestyle changes related to acupuncture theory. For counsellors it was drawn from the humanistic approach, which many contrasted with what was perceived as the “quick fix” of cognitive behavioural therapy (CBT).

*I would like to think that the whole approach is based on longer term change, in terms of the client learning ways of coping. Not just with the present crisis, whatever it might be, but hopefully that they would take something from it that they could apply in the future.*(counsellor1)

When comparing acupuncturists and counsellors, the reported long-term focus involved some differences in approach, as described above, as well as some similarities, especially with regard to encouraging insight into root causes on an individual basis and seeking small incremental changes as important precursors of overall benefit.

### Identifying root causes

The commitment to identifying and addressing the root causes of depression within the treatment process was a commonly expressed factor among both acupuncturists and counsellors. Acupuncturists commonly used the theoretical concept of “root” (ben) and “branch” (biao).

*Within Chinese Medicine we talk about treating the root and the branch. And there is merit in treating both.*(acupuncturist2)

*Looking for underlying causes. Obviously there's some symptomatic stuff, but always looking at the root causes and using that as where I would start to work out my treatment principles.*(acupuncturist3)

The counsellors were more interested in “going deeper” and “further back (in time)” with their clients as a way of getting to the root causes.

*From my experience generally, you've got to get to the root causes of depression, so it's much deeper than a CBT approach, so I think if people don't do that when clients present depression, then perhaps they are failing the clients at some level.*(counsellor2)

*I suppose for me the long term change generally stems from a clear understanding of just what's been at the root of it all.*(counsellor3)

The implications of this approach, as reported by several practitioners, are that unless the underlying causes are addressed, then it is likely that there will not be the sustained long-term change that is desired.

### Individualisation

When practised within the theoretical framework of traditional Chinese medicine, acupuncture treatment has been customised to the individual, such that treatment varies not only between patients but also, for the same patient, it varies over time. Likewise for the counsellors in the trial, all of whom were committed to working within the humanistic tradition, a person-centred approach was provided.

*Everybody's different, so whenever a patient comes in, obviously, you take their full medical history. And quite often there will be other key characteristics, so not everybody gets the same points diagnosis, because of the different underlying features of their condition.*(acupuncturist4)

*That the reason for depression for individuals and how it's processed is unique and therefore the process focuses on how the client experiences it. I also think it's useful to bear in mind the possible causes and theories to be shared with the client.*(counsellor4)

### Valuing incremental change

Practitioners reported a certain amount of caution about expecting sudden or cathartic changes in their patients. More common was a sense that positive benefit required a series of small incremental changes that when combined with self-help approaches would hopefully build a more sustained improvement.

*Well long term change can even come about from even small changes, really. If somebody can just sleep all night, it's surprising.*(acupuncturist5)

*I think to try almost to help people take responsibility and realise that there maybe is something that they can do. I think sometimes people get trapped in feeling depressed and feeling unable to do anything. So almost helping empower them to find changes that they can start to make, to make those improvements for themselves, which over time build up and help them move forward.*(counsellor5)

### Addressing concurrent physical symptoms

Acupuncturists generally stressed the importance of addressing concurrent physical symptoms, for example helping patients relax or sleep better in order to be more receptive to change.

*What I find really interesting about working on the trial is that a lot of time people came with depression but in reality there was a host of other signs and symptoms that they were not happy in their life. Just kind of bowel, musculoskeletal, anything. So when they got a sense that I was treating them as a whole person, rather than just treating the depression..…… When I'd sorted out their back, their stomach, ulcers, bad knees, you know, suddenly their depression got better. To me, that's how Traditional Chinese Medicine should be – it works with everything. Rather than, you know, like depression as a standalone thing.*(acupuncturist2)

By contrast, the counsellors were less focused on the physical symptoms, and worked more on the assumption that by treating the underlying causes of depression there should be a knock-on effect in terms of reducing physical symptoms.

*I'm not particularly interested in just treating symptoms, but what I'm usually interested in and what people are usually asking for is that they want to understand why they're feeling the way that they are. If you can deal with that issue, then it should help them to rid themselves of the symptoms really.*(counsellor6)

### Lifestyle changes

Almost all of the acupuncturists highlighted the importance of giving advice about lifestyle change that was relevant to the Chinese medicine perspective.

*Yeah, it's overall sort of aimed at the long-term and I think people, you know, if they get further along with the acupuncture, they realise that it's all to do with lifestyle change as well and that those things are going to be the ones that bring them lasting benefit. Because often people start to feel better after a few treatments and then when they build in the lifestyle thing, when they actually start to think, oh, this acupuncture seems to be working for me, maybe I'll follow some of the advice as well.* (acupuncturist6)

Those acupuncturists who reported on the role of lifestyle change tended to stress the importance of tailoring the advice to the patient, both in terms of content related to acupuncture theory (e.g. diet, relaxation, exercise, etc) as well as likely acceptability.

The counsellors worked within a trial protocol that had a strong emphasis on working within the humanistic tradition, which included being non-directive. [11] The protocol therefore limited the extent that they could offer lifestyle advice within the trial. Some counsellors, who typically worked more eclectically elsewhere, said they might normally include lifestyle advice, especially when working with people with addictions.

### Therapeutic relationship

Counsellors consistently stressed the importance of the therapeutic relationship.

*I tend to think that lasting change requires a greater degree of insight [than CBT]. But I think the change often comes – I suppose this is what puts me very squarely in the humanistic camp, the existential camp, I think it's often the relationship itself – it's actually having a new experience of a relationship for some people can be transformative.*(counsellor3)

*But the essence of my approach is the relationship and building good levels of rapport and trust and helping the client to feel okay with you. So it's an okay space at the start and if that forms well, that's a springboard, hopefully, for sort of therapeutic practice and change for people.*(counsellor2)

However for some acupuncturists, this was also reported as a component of the treatment process that helped engender longer-term gains.

*And it's kind of like a trust relationship – if they believe in the acupuncture, they believe in what you're telling them. And that kind of gives them the impetus to carry on in the longer term.*(acupuncturist6)

### Careful “pacing” based on “readiness to change”

Many of the counsellors emphasised the need for careful “pacing” such that the process and tools employed were tailored and timed for each individual, depending on the “readiness” to change.

*And what I'm very aware of in counselling is that people will come to therapy at different stages of readiness to change. And it's very difficult to measure how useful it's been. So for example, with one client I can think of in the trial, I think he would have – I think it was probably a first experience for him, from being listened to and from being taken seriously and I think that was huge. He was very stuck. In terms of how much that was going to affect outcomes, in terms of was he going to make the necessary changes, he might have been able to begin to identify, or briefly identify, what it was in his life that was making him depressed – in terms of outcomes he wasn't at the stage where he was going to make any massive changes. But I think just having that experience, that first experience. That kind of thing is so hard to measure, isn't it?*(counsellor6)

*I tend to go with the speed of the client. However, if I can – my personal approach would be to help them in as short a sessions as possible to get them to feel better. However, I'm very careful to work with their speed. So am I short or long term.*(counsellor7)

A number of acupuncturists identified issues related to timing, expressing the point that as patients improve, ideally appointments should be spread out towards the end of the course of treatment and beyond, as a way of sustaining benefit over the longer term.

In summary, the themes capture both similarities and differences in approaches between acupuncture and counselling, with most practitioners of both interventions having a shared sense of the prerequisites for sustained long-term benefit. The impression formed from the interviews is that the various approaches do not operate in isolation. Although the emphasis may vary, the themes appear to be integrated into a coherent combination that uniquely informs the practice of each practitioner.

### Generalisability issues

With regard to the extent that these findings are transferable to other settings, the majority of counsellors said that the severity of the depression amongst trial patients was not markedly dissimilar to the people they routinely saw. By contrast, the acupuncturists were generally less used to dealing with patients with moderate to severe depression, and many said that they less commonly saw patients who consulted primarily because of a diagnosis of depression. Several of the therapists reported that the trial patients had a different attitude towards treatment compared to their private patients, particularly a more limited knowledge of the therapeutic intervention and a lower level of motivation to help themselves. Therapists also noted that there were a greater proportion of patients in lower income brackets, with more long-standing depression, or with unmanaged drug/alcohol addictions.

## Discussion

### Principal findings

Both acupuncturists and counsellors generally delivered treatments with an eye to encouraging longer-term change with the intention that, ideally, any putative benefit would be sustained well beyond the 12 sessions that were provided within the trial. While both types of practitioners reported on the need to address the root causes, the approach differed. For the acupuncturists there was a focus drawn from Chinese medicine theory on treating the root cause (the *ben*) as well as the manifesting symptoms (the *biao*) with the precise details of the intervention customised to their patients at an individual level. Meanwhile the approach of the counsellors was to get below the surface of the clients' problems and to “go deeper” and “further back (in time)” as a way of getting a handle on the root cause and, as with the acupuncturists, this required an individualised approach.

There were further differences in approach to facilitating more sustained benefits. Acupuncturists were more focussed on physical symptoms, on whether these could be resolved by acupuncture in order to speed up the improvements in the symptoms of depression, and on providing lifestyle advice linked to the Chinese medicine diagnosis. Meanwhile counsellors were more explicit on the importance of a strong therapeutic relationship accompanied by a careful consideration of what might be a manageable pace of change.

### Strengths and limitations

The sub-study is nested within the largest trial to date of acupuncture or counselling for depression, and provides unique access to clusters of practitioners who have reported here on the treatments that they provided. The patient group is clearly defined, all with “moderate” to “severe” depression as categorised by the Beck Depression Inventory (BDI-II). The methods used, involving interviews, verbatim transcripts, and thematic analysis, are consistent with many of the markers of quality in qualitative research. [12] We have helped establish the credibility and dependability of the results through involving an independent research team (LN and RC) to help conduct the study, by arranging meetings of stakeholders to review the research questions and the methods used, and by discussing the emerging themes and refining these through debate to agreement. A substantive report emerged from this process, from which a component is reported here. With regard to the transferability of the results, we have provided details of the practitioners as well as the patients who were the focus of our sub-study, such that readers can draw conclusions regarding relevance for other areas. In terms of limitations, our data is limited to patients receiving acupuncture as practised by those using the theories of traditional Chinese medicine and to clients receiving counselling as provided in the humanistic and non-directive and person-centred style.

### Relationship to the wider literature

There is limited evidence in the literature for longer-term benefits associated with acupuncture for depression. [13] In a systematic review of RCTs, counselling for depression has been reported to be no better than GP care in the long term [14], though in a review of pre-post comparisons of client outcomes, benefits have been reported to last a year or more. [15] The trial, within which this sub-study was nested, provided some further evidence on longer-term effects beyond the period of treatment. [4] Within the wider literature there is a dearth of evidence on the acupuncture treatment factors that might be associated with longer-term change in the symptoms of depression. The findings from a small study involving interviews with six practitioners in a trial of acupuncture for back pain found that these acupuncturists had a goal of a positive long-term outcome, and developed a therapeutic partnership to support the active engagement of patients in their own recovery. [16] Consistent with many of the findings we report here, the authors reported that the key elements were: establishing rapport, using an interactive diagnostic process, matching treatment to the patient, and using explanatory models from Chinese medicine to aid a shared understanding and motivate lifestyle changes to reinforce the potential recovery.

In the counselling literature, a qualitative study reported on the long-term effects of counselling, with data drawn from 15 clients who had received counselling from between one and three years previously. [8] The authors' interpretation of the client interview data led them to describe a model of the change process and mechanisms that were perceived as essential to produce lasting benefit. They identified as key elements of the counselling process the active engagement of the client during and between sessions, and the acquisition of a “box of skills” to be built on further after the counselling was finished. Unlike in the current study, in which all clients of counsellors received humanistic counselling, the authors did not identify the counselling approach provided. Another difference between this study and ours, is that all patients in our study had been diagnosed with depression.

### Implications for clinical practice and research

One implication for clinical practice is that, while both acupuncture and counselling for depression appear to be associated with longer-term benefits, the interventions seem to work in different ways. Further research is needed from the perspective of patients and clients on their experiences of treatment from these two modalities. To assist referral, a clearer understanding is needed of which type of person with depression would benefit from acupuncture and which from counselling. While taking into account patient preference, such a typology would provide referring clinicians with valuable guidance on suitability for referral.

Another question is in regard to the nature of depression, and the extent to which physical symptoms are also present. Evidence suggests that 69% of people experiencing depression in primary care initially present with physical symptoms [17] and 50% of people with depression are also in pain. [18] Our study has found that acupuncturists are interested in the physical symptoms that may accompany depression, and will routinely address these symptoms as part of the treatment.

One can speculate that this focus on physical symptoms may in part explain why the acupuncturists in this trial, who reported being less experienced in treating moderate to severe depression, nevertheless delivered just as good outcomes as the counsellors, who encountered this level of depression more commonly in routine practice. Further research is needed into the patient perspective on the treatment of depression with co-morbidities, and specifically the value they place on the comorbid symptoms being addressed concurrently.

Our research raises some important questions about the clinical practice of acupuncturists and counsellors when treating a population with depression when combined with unmanaged drug and alcohol addictions. Further research might be useful to explore appropriate strategies, which might be about ensuring the availability of options, including the involvement of other agencies, and practical support.

## Conclusions

Long-term change appears to be an important focus within the practices of both acupuncturists and counsellors. Practitioners of both interventions generally stressed that, for longer-term benefit, there needs to be an individualised approach with a focus on root causes. Acupuncturists more often emphasised the importance of addressing concurrent physical symptoms and highlighted the importance of relevant lifestyle change. Counsellors commonly stressed the importance of the therapeutic relationship and emphasised the need for appropriate “pacing” such that the timing of the counselling process is geared to the “readiness” to change.

## Supporting Information

Text S1 **Consolidated criteria for reporting qualitative studies (COREQ): 32-item checklist.**
(DOCX)
